# Dynamic Changes and Potential Correlations between Microbial Diversity and Volatile Flavor Compounds in Chinese Medium-Temperature Daqu during Manufacturing

**DOI:** 10.3390/molecules29204851

**Published:** 2024-10-13

**Authors:** Xin Nie, Xiaohan Jia, Kaixian Zhu, Ziqing Ling, Hongfan Chen, Jing Xie, Zonghua Ao, Chuan Song, Caihong Shen, Chenglin Zhu, Wei Yan, Jiabin Wang, Yijing Wang, Zhiping Zhao

**Affiliations:** 1 Culinary Science Key Laboratory of Sichuan Provincial Universities, College of Culinary and Food Science Engineering, Sichuan Tourism University, Chengdu 610100, China; 2 Luzhou Laojiao Co., Ltd., Luzhou 646000, China; 3 College of Food and Biological Engineering, Chengdu University, Chengdu 610106, China; 4 College of Food Science and Technology, Southwest Minzu University, Chengdu 610041, China; 5 Inner Mongolia Hetao Liquor Group Co., Ltd., Bayan Nur 015400, China; 6 School of Liquor-Brewing Engineering, Sichuan University of Jinjiang College, Meishan 620860, China

**Keywords:** Baijiu medium-temperature Daqu, microbial structure, volatile organic compounds, high-throughput sequencing, correlation

## Abstract

To investigate the dynamic changes and potential correlations between microbial diversity and volatile organic compounds (VOCs) during Chinese medium-temperature Daqu (MTD) manufacturing at different key stages, in this study, high-throughput sequencing (HTS) and gas chromatography–ion mobility spectrometry (GC–IMS) were employed to analyze the microbial diversity and VOCs of MTD, respectively. The results showed that *Weissella*, *Staphylococcus*, *Thermoactinomyces*, *Kroppenstedtia*, and *Lactobacillus* were the dominant bacterial genera, while *Aspergillus*, *Alternaria*, *Thermoascus*, *Thermomyces*, *Wickerhamomyces*, and *Saccharomyces* were the dominant fungal genera. A total of 61 VOCs were detected by GC–IMS, among which, 13 differential VOCs (VIP > 1) were identified, that could be used as potential biomarkers to judge the fermentation stage of MTD. *Kroppenstedtia* and *Saccharopolyspora* were positively correlated with 3-methyl-2-butenal and 2,2,4,6,6-pentamethylheptane-D, respectively, and both of these were positively correlated with butanal-D. *Acetobacter*, *Streptomyces*, and lactic acid bacteria (LAB) including *Leuconostoc*, *Pediococcus*, *Weissella*, and *Lactobacillus* were negatively correlated with their associated VOCs, while fungi were generally positively correlated with VOCs. *Wickerhamomyces*, *Saccharomyces*, and *Candida* were positively correlated with butan-2-one-M. This study provides a theoretical basis for explaining the mechanisms of MTD flavor formation and screening functional microorganisms to improve the quality of MTD.

## 1. Introduction

Baijiu, with a complex flavor dominated by esters, is one of the six spirits distilled around the world. It is made from grains, including sorghum, rice, glutinous rice, wheat, and corn through complicated manufacturing process, such as boiling, fermentation, and distillation, with Daqu used as the saccharifying and fermenting agent [1]. Daqu supplies functional microorganisms and complex enzymes to catalyze the biochemical reactions that occur during the fermentation process. Such microorganisms and enzymes can use starch, proteins, and other nutrients as substances, and generate the characteristic flavors of baijiu [2]. Furthermore, the rich flavor substances and precursors in Daqu can accumulate during the fermentation process, which produces abundant flavors in the baijiu through direct and/or indirect ways [3]. Medium-temperature Daqu (MTD) is mainly used for the production of strong-flavor baijiu [4].

Generally, the manufacture of MTD is complicated. Firstly, wheat, other grains, and water are well mixed, followed by grounding and stirring, and the mixture is transferred into brick-shape vessels in order to make brick-shape Daqu [4]. Subsequently, the brick-shape Daqu is stored at room temperature for 30 days, followed by four key stages, namely, the Anqu stage, Peijun stage, fermentation conversion stage, and storage stage. During the Anqu stage, the brick-shape Daqu is stacked and covered with straw mats, which is important for microbial enrichment and aroma substance accumulation [5]. During the Peijun stage, molds grow on the surfaces of the brick-shape Daqu [5]. During the fermentation conversion stage, the temperature is slowly cooled down to room temperature, and the brick-shape Daqu gradually dries [5]. In the storage stage, Daqu is stored for at least three months until it can be used for baijiu brewing.

During baijiu brewing, Daqu enriches a large number of functional microorganisms, such as *Aspergillus*, *Lactobacillus*, *Bacillus*, *Rhizopus*, *Saccharomycetes*, and so on [6]. Kang et al. [7] investigated the distribution of volatile substances in three types of Daqu based on HS-SPME-GC–MS, and a total of 105 volatile compounds were detected, of which, 14 were identified as potential markers for distinguishing different types of Daqu.

Previous studies have focused on the microorganisms and flavor compositions of mature Daqu based on HTS and GC–MS [4,6,8]. However, research on the dynamic changes and correlations of microbial diversity with the volatile compounds of Daqu at different stages is rare. In this study, the microbial communities and VOCs in MTD during the four core fermentation stages (Anqu stage, Peijun stage, fermentation conversion stage, and storage stage) were explored based on HTS and GC–IMS. Furthermore, the correlations between dominant microorganisms and characteristic VOCs were investigated. This study promotes further elucidations of the potential correlations between microorganisms and VOCs during MTD processing, as well as the utilization of functional microorganisms in MTD.

## 2. Results and Discussion

### 2.1. Qualitative Analysis of the VOCs in MTD

**Figure 1:**
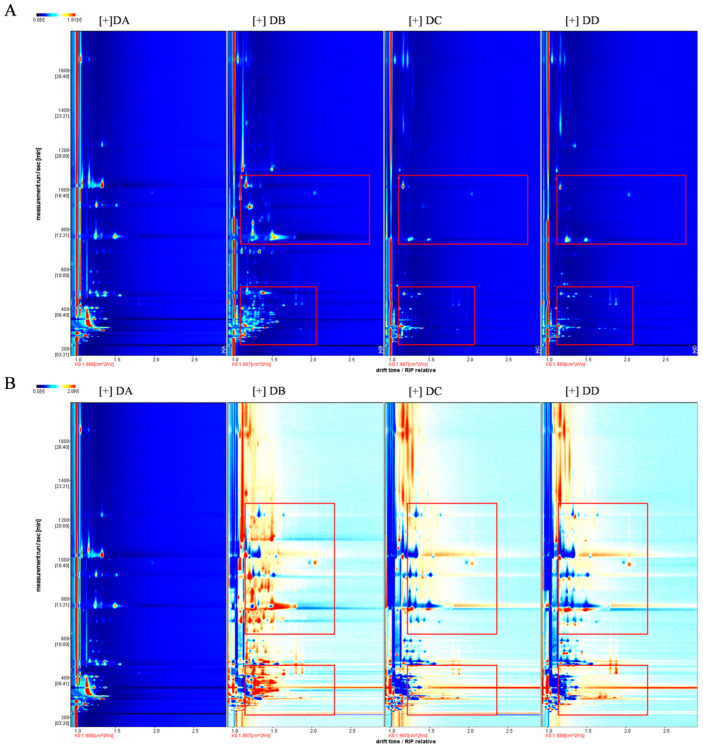
VOCs identified by GC–IMS for the four stages of MTD samples. 2D topography (**A**) and the differences in a comparison plot with DA as reference (**B**). Red boxes represent VOCs with significant variations.

**Figure 2:**
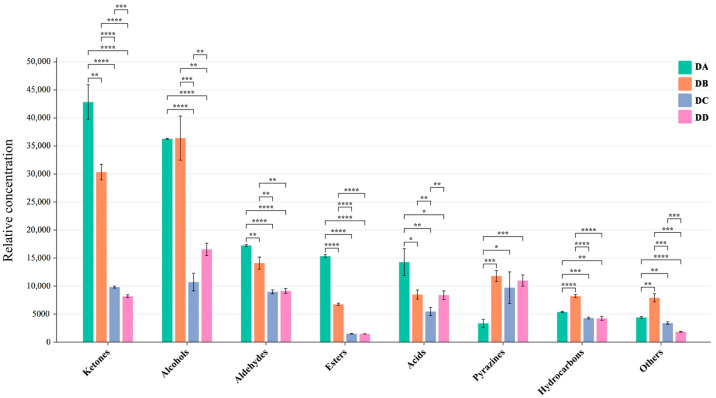
The contents of different VOCs in DA, DB, DC, and DD samples (* *p* < 0.05, ** *p* < 0.01, *** *p* < 0.001, **** *p* < 0.0001).

The DA (Anqu stage), DB (Peijun stage), DC (fermentation conversion stage), and DD (storage stage) samples had a similar composition of VOCs with different contents, as seen in Figure 1. A qualitative analysis of VOCs was conducted using retention time and ion migration time, while quantitative analysis was performed using IMS system peak volume. Sixty-one peaks were identified using the NIST 2014 and IMS databases, with fifty peaks having corresponding matching information. The 50 known VOCs included 12 alcohols, 10 ketones, 8 aldehydes, 7 esters, 5 hydrocarbons, 2 acids, 2 pyrazines, and 4 others, as shown in Table S1. The quantitative relationship between the different types of VOCs is shown in Figure 2.

Alcohols are primarily derived from lipid oxidation and yeast fermentation [9]. Microecological markers, such as 1-hexanol, 1-pentanol, 2-methyl-1-propanol, 3-methylbutanol, and butan-2-ol, are common alcohol components in baijiu, which play important roles in the quality of MTD and in the flavor formation of baijiu [10]. The production of 2-methyl-1-propanol, derived from the metabolism of by *Pichia kudriavzeviiin* in Daqu, is influenced by the fermentation temperature. DB exhibited the highest level of 2-methyl-1-propanol, which was probably due to the elevated temperatures and increased enzyme activity during the Peijun phase [11]. The concentrations of saturated alcohols in DA and DB were significantly higher than in DC and DD, which might be related to the higher expression of key enzymes involved in MTD (such as alcohol dehydrogenases and aryl-alcohol dehydrogenase) during the pre-fermentation phase [12]. Butanol, a key aroma compound in strong-flavor baijiu, imparts a banana-like odor characteristic in baiju’s flavor profile [13]. Song et al. [14] identified butanol as a marker compound that can distinguish strong-flavor baijiu from different regions.

Aldehydes and ketones have significant effects on the aroma characteristics of food. Wu [15] found that aldehydes in Daqu were generated below 50 °C. During the Anqu stage, lower temperature led to a significant enrichment of aldehydes in the DA group. A previous study revealed that the concentrations of certain ketones, such as heptan-2-one, 3-penten-2-one, cyclopentanone, and 3-hydroxy-2-butanone, were significantly higher in strong-flavor baijiu produced in the Sichuan region compared to the Jianghuai region. These compounds are considered characteristic aroma markers of strong-flavor baijiu from Sichuan [13].

Most esters in baijiu are generated through the reaction of alcohols with coenzyme A, catalyzed by esterases, which are closely related to microbial metabolism, primarily involving yeasts and molds [16]. Esters contribute to the pleasant odors of Daqu, such as fruity, floral, pineapple, apple, and banana aromas. The main esters detected in Daqu were butanoic acid propyl ester, methyl butyrate, ethyl 3-methylbutanoate, and ethyl acetate. The ester content in the DA group was significantly higher than in the DB, DC, and DD groups, which was probably due to differences in fermentation temperature, microbial diversity, and oxygen concentration at various stages [17,18]. It has been reported that higher fermentation temperatures lead to a significant loss of esters [14]. Additionally, the DA group produced more short-chain esters, attributed to lower temperatures and higher moisture content, in agreement with previous findings [14].

Pyrazines, key flavor compounds in baijiu, contribute barbecue, creamy, nutty, and toasty aromas to MTD [19]. Among the pyrazines detected in the MTD samples, 2-Ethyl-6-methylpyrazine and 2,5-dimethylpyrazine were predominant. The concentration of pyrazines was significantly influenced by temperature, with the DB group showing the highest levels. Pyrazine production is largely driven by the release of ammonia through the pyrolysis of amino sources [20]. Additionally, pyrazines are formed via the Maillard reaction at high temperatures [11], with optimal production occurring between 50 °C and 60 °C [15].

### 2.2. Fingerprint Analysis of VOCs

**Figure 3:**
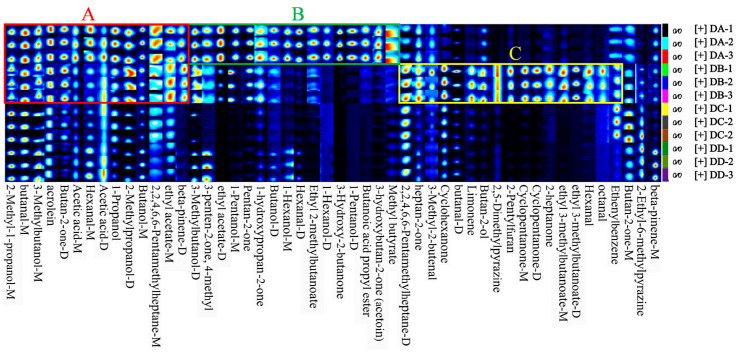
Fingerprints of VOCs in DA, DB, DC, and DD samples of MTD. The A–C boxes represent the enriched VOCs in each sample.

All samples were analyzed in triplicate: DA (DA-1, DA-2, DA-3), DB (DB-1, DB-2, DB-3), DC (DC-1, DC-2, DC-3), and DD (DD-1, DD-2, DD-3). As shown in Figure 3, area A indicates that the concentration of VOCs in the DA and DB group was significantly higher than in the DC and DD group, including 2-methyl-1-propanol, butanal, 3-methylbutanol, acrolein, butan-2-one, 1-propanol, 2-methylpropanol. Most of these compounds have low detection thresholds and strong aromas, making the DA and DB stages crucial for the flavor formation of MTD. Area B shows that VOCs were more concentrated in the DA group compared to the DB, DC, and DD groups, including 3-methylbutanol, 3-penten-2-one, 4-methyl, ethyl acetate, 1-pentanol, pentan-2-one, 1-hydroxypropan-2-one, and 1-hexanol. Esters were particularly abundant in the DA group, likely due to differences in microbial metabolic activity influenced by the temperature and manufacturing stages [21]. Among these, ethyl acetate, butanoic acid propyl ester, and ethyl 2-methylbutanoate are common esters in strong-flavor baijiu, contributing a pineapple, floral, and banana-like aromas, respectively [22]. Aera C suggests that VOC concentrations were higher in the DB group than in the DA, DC, and DD groups, mainly including heptan-2-one, cyclohexanone, butan-2-ol, 2,5-dimethylpyrazine, cyclopentanone, 2-heptanone, ethyl 3-methylbutanoateand and others. The DB group produced higher levels of aldehydes and ketones, which are produced though lipid oxidation and the synthesis of flavor precursors by microorganisms such as *Kroppenstedtia*, *Thermoactinomyces*, and *Bacillus* [6]. These microorganisms thrive in high temperatures (>60 °C) and low-moisture conditions (10–15%), which facilitated the production of aldehydes and ketones in the DB group [6].

### 2.3. Multivariate Statistical Analysis of the VOCs

**Figure 4:**
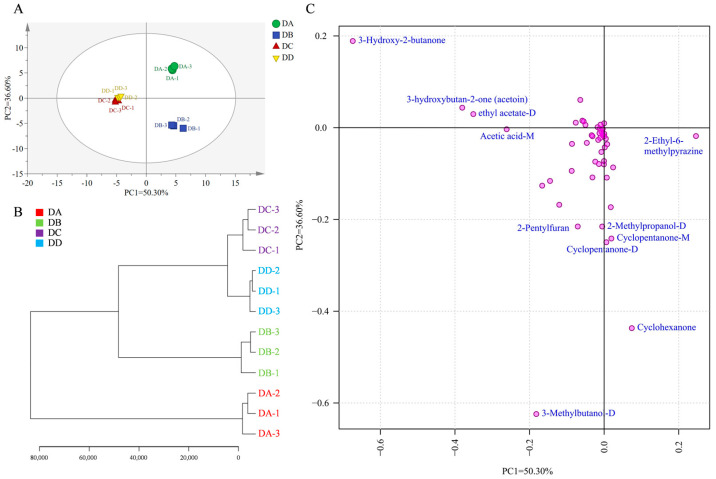
Multivariate statistical analysis of the VOCs in DA, DB, DC, and DD. Plot of PCA scores (**A**), clustering results (**B**), and loadings (**C**).

To further investigate the differences between groups, multivariate statistical analysis of the VOCs at different stages of MTD was performed using principal component analysis. The contributions of PC1 and PC2 were 50.30% and 36.60%, respectively, with a cumulative contribution of 86.90% (Figure 4A). The DA and DB groups were significantly separated along the second principal component, indicating the significant differences in VOCs between these two groups. The DC and DD groups were more similar to each other, and were clearly separated from the DA and DB groups along the first principal component. These samples were clustered into three groups (Figure 4B), reflecting the VOC variations at different fermentation stages. Most VOCs are formed through interactions between microorganisms and their environment. As the microbial structure changed only slightly during the later stages of MTD fermentation [23], the DC and DD groups clustered together, distinguishing them from the DA and DB groups. R2X (cum), the main parameter for evaluating the PCA model, showed a value above 0.5, indicating good model interpretability. The contributions of the compounds related to the principal components are shown in Figure 4C. The flavor contributing most to the first principal component was 3-hydroxy-2-butanone, followed by 3-hydroxybutan-2-one (acetoin), ethyl acetate-D, acetic acid-M, and 2-ethyl-6-methylpyrazine. For the second principal component, 3-methylbutanol-D was the most significant contributor. Other compounds, including cyclohexanone, cyclopentanone-D, cyclopentanone-M, 2-methylpropanol-D and 2-pentylfuran, also made significant contributions to the second principal component.

### 2.4. Analysis of the Differential VOCs in MTD

The variable importance in projection (VIP) values based on the OPLS-DA model were employed to identify differential VOCs in MTD, as shown in Table 1. A total of 13 differential VOCs were identified, including four alcohols (butanol-M, 1-hexanol-M, 3-methylbutanol-M, 1-propanol), three hydrocarbons (β-pinene-M, 2,2,4,6,6-pentamethylheptane-M, 2,2,4,6,6-pentamethylheptane-D), two aldehydes (3-methyl-2-butenal and butanal-D), one ketone (3-penten-2-one, 4-methyl), and three others (ethenylbenzene, butan-2-one-M, butan-2-one-D).

**Figure 5:**
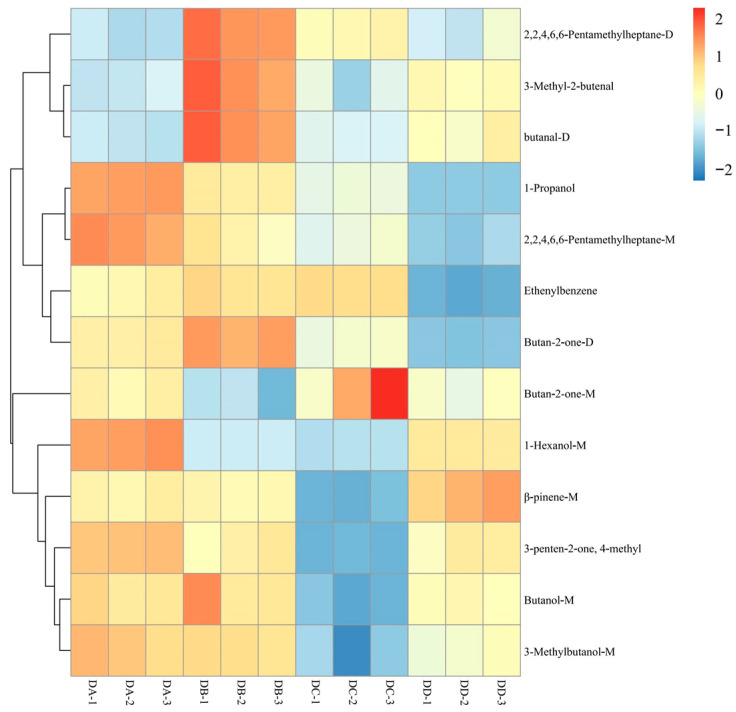
Hierarchical clustering analysis of the differential VOCs in DA, DB, DC, and DD samples.

To better assess the differences in the 13 differential VOCs of DA, DB, DC, and DD MTD, their relative contents were analyzed using hierarchical clustering analysis, as shown in Figure 5. Three alcohols (1-hexanol-M, 3-methylbutanol-M, and 1-propanol) were predominantly enriched in the DA group, which may be related to the enzyme activities of MTD at different stages [11]. Furthermore, 1-hexanol-M and 3-methylbutanol-M produce banana and brandy odors, respectively, and are generally derived from the degradation of amino acids via the Ehrlich pathway [24]. These two alcohols are the key odorants in strong-flavor baijiu, and their accumulation during the early stages of Daqu processing contributes to the rich aroma profile of baijiu [25].The two aldehydes (3-methyl-2-butenal and butanal-D) were more abundant in the DB group than in the other groups, which may be due to lipid oxidation and thermal decomposition [26]. β-pinene-M was the only compound found in the highest concentration in the DD group, which was synthesized primarily via the Mevalonate pathway (MEP) from acetyl coenzyme A and pyruvate lyase, supplied by Daqu plastids and carbohydrate pools in the cytoplasm [27]. β-pinene tended to accumulate at higher levels toward the end of fermentation, with the consumption of carbohydrates, which is in agreement with a previous study [28]. Terpenes are widely known for their strong aromatic qualities and the accumulation of β-pinene (rosin) provides an excellent flavor basis for strong-flavor baijiu [28].

### 2.5. α-Diversity Analysis of MTD Based on HTS

**Figure 6:**
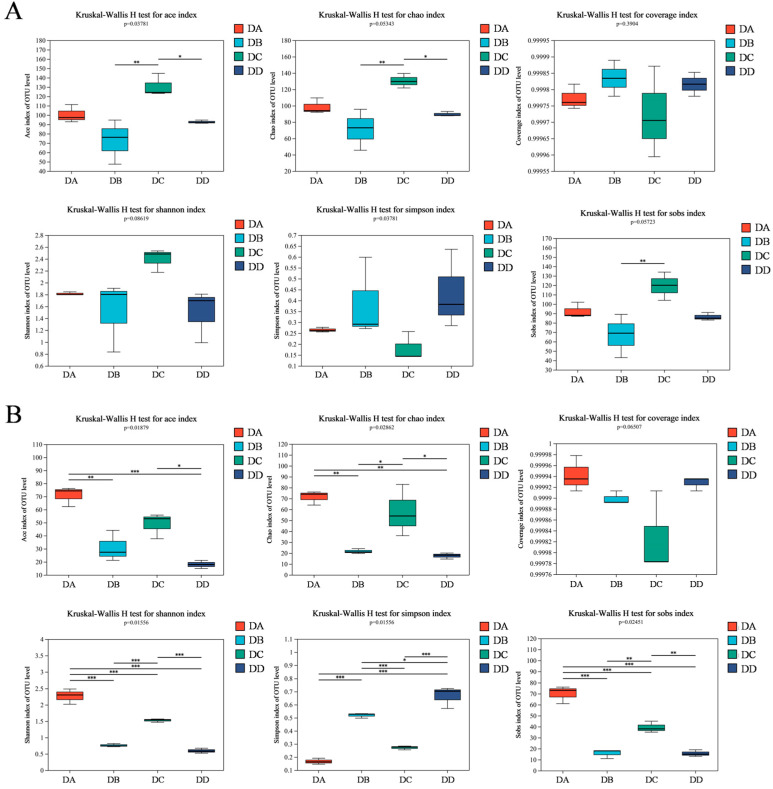
The α-diversity indices of bacteria (**A**) and fungi (**B**) in the DA, DB, DC, and DD samples (* *p* < 0.05, ** *p* < 0.01, *** *p* < 0.001).

The α-diversity analysis of microorganisms in MTD is shown in Figure 6 (Tables S2 and S3). The ACE, Chao1, Sobs, and Shannon indices indicate that the diversity and homogeneity of bacteria in the DC group were significantly higher than in the other groups. The Simpson index in the DD group was the highest, indicating that a few bacterial species became dominant. Microbial diversity in MTD during the storage stage (the DD group) was reduced because of the changes in temperature and moisture levels [29]. In contrast, the DA group had a high abundance and homogeneity in fungi, as shown in Figure 6B. The Simpson index for fungi was also higher in the DD group, with a few fungi species becoming dominant after a series of environmental changes. The Coverage indices for bacteria and fungi are shown in Figure 6A,B, respectively, while the Shannon dilution curves for both bacteria and fungi are shown in Figure S1.

### 2.6. Analysis of Microbial Community Structure in MTD

**Figure 7:**
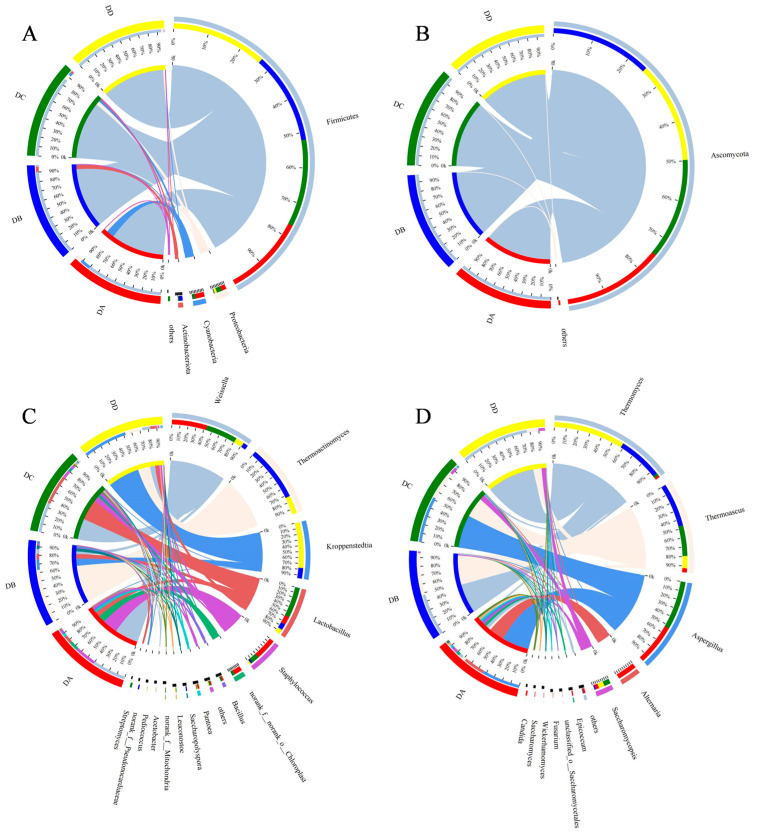
Relative abundance of bacteria at the phylum level (**A**) and genus level (**C**), and fungi at the phylum level (**B**) and genus level (**D**) in MTD. Species with relative abundance below 1% were defined in the samples as “Others”.

Species abundance and community composition were analyzed at the phylum and genus levels, as shown in Figure 7. Species with an average relative abundance > 1.0% were classified as dominant, and those below 1.0% were categorized as others.

Firmicutes, Proteobacteria, Cyanobacteria, and Actinobacteriota were the dominant bacterial phyla (Figure 7A). The abundance of Firmicutes in all samples exceeded 80%, indicating that Firmicutes was the dominant bacterial phylum in MTD, which is similar to a previous study [3]. At the genus level, a total of 15 dominant genera were identified (Figure 7C), including *Weissella*, *Thermoactinomyces*, *Kroppenstedtia*, *Lactobacillus*, *Staphylococcus*, and others. The predominant bacterial genera in the DA group were *Weissella* (41%) and *Staphylococcus* (26%). *Weissella* is generally the dominant bacterial genus in the pre-fermentation stage of Daqu. During fermentation, the microorganisms continuously produce saccharolytic enzymes, which can degrade starch into reducing sugars, which are further converted into lactic acid by *Weissella* through the pyruvate metabolic pathway [30]. During the Peijun stage (the DB group), temperatures rose to their peak, the heat-resistant flora greatly proliferated, and they influenced the enzyme activity and VOC formation [31]. *Thermoactinomyces* (65%) and *Kroppenstedtia* (12%) were the dominant bacterial genera in the DB group, and the extended high-temperature period sustained these heat-resistant microorganisms. *Thermoactinomyces* increased the activity of heat-stabilizing enzymes and promoted the Maillard and caramelization reactions, which contributed to the formation of black or dark brown compounds on the surface of the MTD [32]. This was consistent with the GC–IMS results showing that pyrazines had a higher content in the DB group. *Kroppenstedtia*, a high-temperature-resistant actinomycete, is a marker microorganism of high-temperature Daqu and is closely related to the synthesis of multiple flavor compounds [32]. The relative abundance of both *Lactobacillus* and *Weissella* increased to 36% in the DC group. *Lactobacillus* has been defined as a biomarker for bacterial community succession during the fermentation of strong-flavor baijiu and dominates in the late stage of Daqu fermentation [33]. There were 11 bacterial genera in the DC group with abundances of more than 1%, with more dominant genera than the other groups, aligning with the results of the α-diversity analysis. *Kroppenstedtia* (54%), *Thermoactinomyces* (21%), *Weissella* (10%), and *Lactobacillus* (5.6%) were the dominant bacterial genera in DD group. During the storage period, stress-resistant microorganisms became the core flora of MTD, shaped by high temperatures, moisture volatilization, and changes in oxygen concentration.

At the phylum level, Ascomycota was the predominant fungal phylum (Figure 7B), while at the genus level, eleven dominant genera were identified (Figure 7D). The DA group had 11 dominant fungal genera (>1%), which was consistent with the α-diversity analysis. *Aspergillus* (45%) and *Alternaria* (24%) were the main dominant genera in the DA group. In addition, the abundance of *Epicoccum*, *Fusarium*, *Wickerhamomyces*, *Saccharomyces*, and *Candida* in DA group was higher compared to the other three groups. The niche breadth of fungal communities in Daqu is narrower than its of the bacterial communities, suggesting that fungi are less adaptable to environmental changes [34]. In the fermentation process of strong-flavor baijiu, the earliest discovered ester-producing yeasts were *Candida* and *Wickerhamomyces*. These fungi produce intracellular esterifying enzymes that influence aroma component production [35]; this could explain why the ester content in the DA group was significantly higher than in the other three groups. During the DA stage, *Candida* and *Wickerhammyces* play an important role in the formation of aroma in Daqu and ester production in baijiu [35]. During the high temperature fermentation stage (the DB group), *Thermoascus* and *Thermomyces* gradually became the dominant fungal genera due to their heat resistance. These fungi can effectively accelerate the decomposition and utilization of starch in Daqu by producing a variety of thermophilic glycoside hydrolases, facilitating saccharification and the conversion of raw materials for the Daqu [30]. After the high-temperature fermentation (the Peijun stage), the abundance of *Aspergillus* increased to 54% in the DC group, which was able to produce extracellular enzymes such as acid/alkaline proteases, leading to the generation of secondary metabolites [36]. It has been reported that *Thermoascus*, *Saccharomycopsis*, and *Aspergillus* are significantly associated with the various biochemical properties of Daqu, including its liquifying, saccharifying, esterifying, and fermenting powers, These fungi also produce hydrolytic enzymes that play important roles in the quality of Daqu [37]. In the storage stage (the DD group), *Thermomyces* and *Thermoascusonce* once again became the dominant fungi, with a total abundance exceeding 90%. These two thermophilic fungi produce enzymes that degrade carbohydrates and form flavor compounds on the surface of Daqu, providing essential flavor base for strong-flavor baijiu [32].

### 2.7. Analysis of LEfSe Differences in Microorganisms

**Figure 8:**
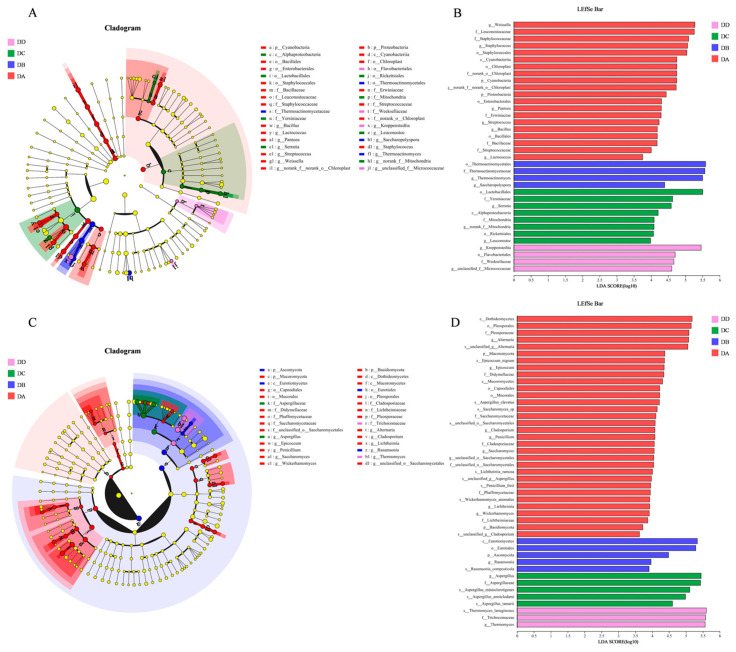
LEfSe and LDA discrimination analysis of dominant microbial flora in MTD. (**A**) LEfSe analysis for bacteria, (**B**) LDA discrimination results for bacteria, (**C**) LEfSe analysis for fungi, (**D**) LDA discrimination results for fungi.

LEfSe differential analysis was conducted between each pair of groups, as shown in Figure 8. The significantly enriched bacterial genera in four stages of MTD are presented in Figure 8A. Specifically, *Weissella*, *Staphylococcus*, *Pantoea*, *Streptomyces*, *Bacillus*, and *Lactococcus* were dominant in DA. *Thermoactinomyces* and *Saccharopolyspora* were the dominant bacterial genera in DB. Meanwhile, *Serratia* and *Leuconostoc* were dominant in DC. *Kroppenstedtia* was the dominant bacterial genera in DD.

The dominant fungi genera in Daqu are shown in Figure 8C, which identifies the key genera at each stage: DA included *Alternaria*, *Epicoccum*, *Cladosporium*, *Penicillium*, *Saccharomyces*, *Lichtheimia*, and *Wickerhamomyces*; DB was dominated by *Rasamsonia*; DC by *Aspergillus*; and DD by *Thermomyces*. These microorganisms can be used as potential biomarkers for distinguishing the different fermentation stages of MTD. The species significantly enriched at each stage are visualized by LDA in Figure 8B and Figure 8D, respectively.

*Pantoea*, participating in the transport and metabolism of carbohydrates and amino acids, plays a role in lipid biosynthesis and VOC formation [38]. *Saccharomycesas* and non-Saccharomyces yeasts (such as *Wickerhamomyces*) are strong ethanol producers. They contribute to the production of flavor compounds through the activity of alcohol dehydrogenase [39]. As a representative of filamentous fungi, *Aspergillus* produces hydrolases that degrade starch into fermentable sugars [40]. Moreover, it is considered the key microorganism in the formation of alcoholic flavor compounds such as esters, pyrazines, and some aromatic compounds [38]. In the later fermentation stage, the fermentation substrates were converted into alcohol and other metabolites, while the sugars in the Daqu were transformed into acids. *Aspergillusis*, with its tolerance to alcohol and acidic environments [37], became more abundant in the DC group. During the storage period (DD group), the significantly enriched bacteria and fungi genera were *Kroppenstedtia* and *Thermomyces*, respectively—both heat-resistant species that produce heat-stabilizing enzymes. These enzymes contribute to the Maillard reaction in Daqu and the formation of aroma compounds. The high-temperature fermentation process results in varying abundances of thermophilic flora at different stages [41]. Consequently, bacteria and fungi were the most enriched species in the DA group and the lowest abundance species in the DD group.

### 2.8. Core Flora Analysis Based on OTU Levels

**Figure 9:**
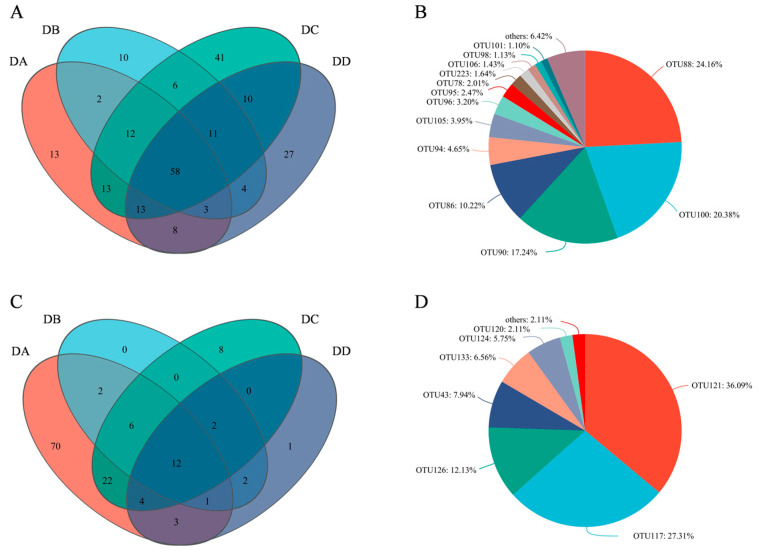
Statistics of core OTUs in the four groups of MTD. (**A**) The number of bacteria; (**B**) The number of fungi; (**C**) The core bacteria with relative abundance >1.0%; (**D**) The core fungi with relative abundance >1.0%.

The biological information and relative abundance of core OTUs with a relative abundance > 1% in MTD are listed in Table S4. Veen diagram analysis (Figure 9A,C) showed 58 core OTUs for bacteria and 12 for fungi. A total of 13 core OTUs of the bacteria had a relative abundance > 1%, as shown in Figure 9B, including *Weissella*, *Thermoactinomyces*, *Kroppenstedtia*, *Staphylococcus*, norank_f_norank_o_Chloroplast, *Pantoea*, *Bacillus*, *Leuconostoc*, and five species of *Lactobacillus*. *Weissella* (24.16%) and *Thermoactinomyces* (20.38%) accounted for the largest proportions, followed by *Kroppenstedtia* (17.24%) and *Staphylococcus* (10.22%). The abundance of *Weissella* suggests its key role in maintaining flavor diversity and microbial community homeostasis. On the other hand, *Weissella* is commonly found in various types of Daqu [42]. Seven of the 12 fungal core OTUs had a relative abundance of more than 1%, including *Thermomyces*, *Thermoascus*, *Alternaria*, *Saccharomycopsis* and three species of *Aspergillus*, as shown in Figure 9D. *Thermomyces*, *Thermoascus* and *Aspergillus* reached abundances of 36.09%, 27.31%, and 22.18%, respectively, and are known for secreting enzymes (such as glucoamylase, cellulase, and thermophilic glycoside hydrolases) to produce abundant VOCs [11]. The presence of *Aspergillus*, *Thermomyces*, and *Thermoascus* at all stages can be attributed to their strong resistance to stress [30,37].

### 2.9. Potential Correlations between Microbial Community and VOCs

**Figure 10:**
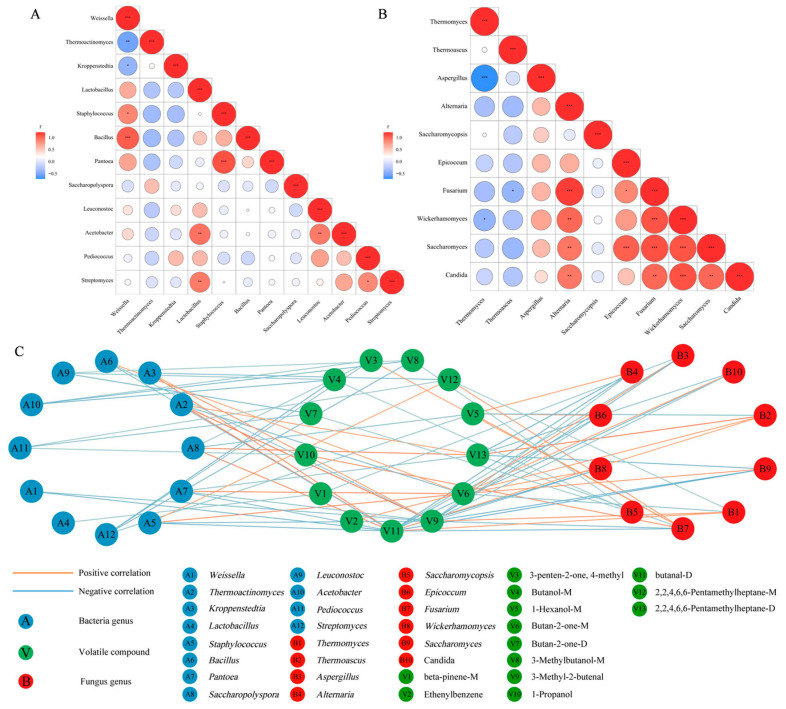
Intragroup correlation of the dominant bacterial genera (**A**) and the dominant fungal genera (**B**), as well as the potential correlations between microbial flora and VOCS (**C**) (* *p* < 0.05, ** *p* < 0.01, *** *p* < 0.001).

Twelve dominant bacterial genera (excluding the unclassified) and 10 dominant fungal genera (excluding one unclassified) were employed for the intragroup correlation analysis, as shown in Figure 10. Red and blue circles represent positive and negative correlations, with the size and color intensity of the circles indicating the strength of the correlation (* *p* < 0.05, ** *p* < 0.01, *** *p* < 0.001). The bacterial intragroup correlations are shown in Figure 10A. *Weissella* was significantly and negatively correlated with *Thermoactinomyces* and *Kroppenstedtia*, while showing a significant positive correlation with *Staphylococcus* and *Bacillus*, due to the two bacteria being more sensitive to temperature. This suggests that the interaction mechanisms of *Weissella* in microbial communities were strongly influenced by temperature [4]. *Acetobacter* exhibited a strong positive correlation with the lactic acid bacteria (LAB) *Lactobacillus* and *Leuconostoc*, both of which are acid-tolerant, which probably promotes their synergistic relationship during fermentation [43]. *Pantoea* showed a positive correlation with *Staphylococcus*, potentially facilitated by fructose produced through starch degradation in Daqu [44]. Fungi exhibited closer correlations compared to bacteria, as shown in Figure 10B. *Aspergillus* had a significant negative correlation with *Thermomyces*. On the other hand, *Alternaria* and *Fusarium* showed strong positive correlations with most of the *Saccharomycetes* (such as *Wickerhamomyces*, *Saccharomyces*, and *Candida*). It has been reported that the populations of *Alternaria* and yeast both increase during acetic acid fermentation [45].

The enrichment of 13 VOCs with VIP > 1 in each sample was as follows: DA (1-propanol, 2,2,4,6,6-pentamethylheptane-M, 1-hexanol-M, 3-penten-2-one, 4-methyl, 3-methylbutanol-M), DB (2,2,4,6,6-pentamethylheptane-D, 3-methyl-2-butenal, butanal-D, butan-2-one-D, butanol-M), DC (ethenylbenzene, butan-2-one-M), and DD (β-pinene-M). The potential relationships between the dominant microbial flora and characteristic VOCs at different stages of Daqu processing were investigated using Spearman’s correlation analysis (|R| > 0.6, *p* < 0.05), as shown in Figure 10C. The orange and blue lines represent positive and negative correlations, with the color intensity representing the strength of the correlation. *Acetobacter*, *Streptomyces*, and LAB (*Leuconostoc*, *Pediococcus*, *Weissella*, and *Lactobacillus*) negatively correlated with the associated VOCs. The 13 VOCs mostly decreased in the DC group, which is consistent with findings that increased in LAB accelerates the microbial succession rate, thus slowing the microbial enrichment and the production of flavor compounds [33]. *Kroppenstedtia* and *Saccharopolyspora*, which are important for baijiu flavor formation [46], showed significant positive correlations with 3-methyl-2-butenal and 2,2,4,6,6-pentamethylheptane-D, respectively, and they both positively correlated with butanal-D. *Staphylococcus* had a positive correlation with butan-2-one-M (R = 0.748, *p* < 0.05), suggesting its involvement in the alcohol metabolism pathway during macrobiotic fermentation by metabolizing substrates to produce volatile ketones, such as butan-2-one-M [47].

Compared to bacteria, fungi generally showed positive correlations with VOCs. *Aspergillus* positively correlated with butan-2-one-M (R = 0.683, *p* < 0.05), probably due to its high levels of ester synthase, which can utilize ketones, aldehydes, alcohols, and acids as substrates to generate esters [21]. *Wickerhamomyces*, *Saccharomyces*, and *Candida* displayed positive correlations with butan-2-one-M and negative correlations with butanal-D. During fermentation, yeasts participate in carbon metabolism pathways to produce aldehydes and ketones, with environmental changes promoting or inhibiting the metabolism of these compounds [4]. *Thermomyces* positively correlated with 3-methyl-2-butenal (R = 0.699, *p* < 0.05) and butanal-D (R = 0.664, *p* < 0.05). *Thermoascus* positively correlated with ethenylbenzene (R = 0.629, *p* < 0.05), butanal-D (R = 0.601, *p* < 0.05), and 2,2,4,6,6-pentamethylheptane-D (R = 0.853, *p* < 0.01). These two thermotolerant bacteria can produce various VOCs by decomposing starch, providing the material basis for the metabolism of other strains and activating the entire flavor metabolic network [23].

## 3. Materials and Methods

### 3.1. Collection of MTD

MTD samples were collected from a baijiu production enterprise located in Luzhou city, Sichuan province, China (105° E, 28° N). The samples were collected at four core stages of Daqu production: Anqu, Peijun, transformation fermentation, and storage stage. Three MTD blocks were randomly selected and labeled as DA (Anqu stage), DB (Peijun stage), DC (transformation fermentation stage), and DD (storage stage), respectively. All samples have three duplicates, termed DA (DA-1, DA-2, DA-3), DB (DB-1, DB-2, DB-3), DC (DC-1, DC-2, DC-3), and DD (DD-1, DD-2, DD-3). The DA, DB, DC, and DD samples were grinded and stored at −20 °C for further analysis.

### 3.2. Detection of VOCs in MTD by GCIMS

GC–IMS analysis was performed in a FlavourSpec^®^ from G.A.S. (Gesellschaft für Analytische Sensorsysteme mbH, Dortmund, Germany) equipped with a syringe and an autosampler unit for headspace analysis. After 15 minutes of incubation at 40 °C, 500 μL of the headspace content was automatically injected by the heated syringe (85 °C). Chromatographic separation was performed on an MXT–1 capillary column (15 m × 0.53 mm) at 60 °C, with nitrogen (N_2_) as the carrier gas (purity ≥ 99.999%) at the following flow rate: 2 mL/min for 2 min, linearly increased to 10 mL/min for 3 min, then to 15 mL/min for 10 min, further to 50 mL/min for 5 min, and finally to 100 mL/min for 10 min. The total GC run time was 30 minutes. After separation in the capillary column, the headspace content was first injected into the ionization chamber for ionization, then through the shutter grid into the drift zone, and finally into the IMS detector. The drift tube was 98 mm in length and maintained at 45 °C. The drift gas (N_2_, purity ≥ 99.999%) flow was set at 150 mL/min. The experiment was carried out in triplicate.

### 3.3. DNA Extraction and Sequencing

The genomes of microorganisms in the MTD samples were extracted using the E.Z.N.A.^®^ soil DNA Kit (Omega Bio-tek, Norcross, GA, USA). The mass of extracted DNA was determined using 1% agarose gel electrophoresis, and the NanoDrop^®^ ND-2000 Spectrophotometer (Thermo Scientific Inc., Waltham, MA, USA) was used to determine the concentration and purity. The 338F (5′-ACTCCTACGGGGAGGCAGCAG-3′) and 806R (5′-GGACTACHVGGGGTWTCTAAT-3′) primers were used to amplify the V3–V4 region of the bacterial 16S rDNA gene. ITS1-F (5′-CTTGGTCATTTAGAGAGGAAGTAA-3′) and ITS2 (5′-GCTGCGTTCTTCATCGATGC-3′) were used to amplify the ITS1–ITS2 region of the fungus. The samples were sent to Majorbio Bio-Pharm Technology Co. Ltd. (Shanghai, China) for sequencing analysis using the Illumina MiSeq PE300 platform (Illumina, San Diego, CA, USA).

### 3.4. Data Analysis

Data processing was performed using Microsoft office 2021. Principal component analysis (PCA) and orthogonal partial least squares discrimination analysis (OPLS-DA) were performed using SIMCA 14.1 (Umetrics, Umea, Sweden). Qualitative analyses of VOCs were carried out using GC–IMS system software VOCal 2014, based on the retention time and drift time. Two-dimensional spectra of VOCs were automatically generated using the built-in Reporter plug-in of the GC–IMS system. The fingerprints were obtained using the built-in Gallery Plot. Cluster dendrograms, hierarchical clustering heatmaps, and VOC peak volume histograms were generated using the R Programming Language (4.2.3, R Foundation for Statistical Computing, Vienna, Austria). Microbial diversity analyses such as α-diversity, dilution curves, microbial community composition, LEfSe analysis, and core OUT analysis were performed on the Major BioCloud platform (https://cloud.majorbio.com). Correlation heatmaps and network diagrams were produced by the Tutu Cloud Platform (https://www.cloudtutu.com) and Cytoscape 3.9.1 (National Institutes of Health, Palo Alto, CA, USA), respectively.

## 4. Conclusions

In this study, the microflora, VOCs, and their correlations were investigated in MTD across different processing stages. A total of 61 VOCs was detected using GC–IMS, of which 50 VOCs were identified, including 12 alcohols, 10 ketones, 8 aldehydes, 7 esters, 5 hydrocarbons, 2 acids, 2 pyrazines, and 4 others. Significant differences in VOCs were observed across the various stages of Daqu processing. Thirteen differential VOCs were screened based on VIP > 1, which could be used as potential biomarkers for distinguishing the tested MTD at different processing periods. High-throughput sequencing (HTS) results suggested that there were 15 dominant bacterial and 11 dominant fungal genera. Spearman’s correlation analysis revealed that microbial diversity was closely related to the formation of VOCs during MTD processing (|R| > 0.6, *p*< 0.05). In the future, the comparison of VOCs profiles at various stages of Daqu production with those of the final product after distillation will be performed. The analysis of the potential relationships between specific microorganisms and VOC profiles will also be further confirmed. Additionally, metagenomics, metatranscriptomics, metabolomics, and other techniques will be employed to investigate the functional microorganisms and their contributions to the production of metabolites and VOCs throughout the MTD processing stages.

## Figures and Tables

**Table 1:** VIP values of differential VOCs of MTD based on OPLS-DA analysis.

	CAS	Flavor Substance	VIP	Aroma Descriptions
1	127-91-3	β-pinene-M	1.46287	Pine, Wood
2	287399-41-1	Ethenylbenzene	1.38991	--
3	141-79-7	3-Penten-2-one, 4-methyl	1.19342	Honey
4	71-36-3	Butanol-M	1.12958	Fruit
5	111-27-3	1-Hexanol-M	1.1228	Banana, Flower, Grass
6	78-93-3	Butan-2-one-M	1.06204	Fragrant, Fruit, Pleasant
7	78-93-3	Butan-2-one-D	1.05515	Fragrant, Fruit, Pleasant
8	123-51-3	3-Methylbutanol-M	1.05106	Burnt, Brandy
9	107-86-8	3-Methyl-2-butenal	1.04748	Almond, Roasted
10	71-23-8	1-Propanol	1.04289	Alcohol, Candy, Pungent
11	123-72-8	Butanal-D	1.04221	Banana, Green, Pungent
12	13475-82-6	2,2,4,6,6-Pentamethylheptane-M	1.03767	--
13	13475-82-6	2,2,4,6,6-Pentamethylheptane-D	1.01351	--

## Data Availability

Data will be made available upon request.

## Supplementary Materials

The following supporting information can be downloaded at: https://www.mdpi.com/article/10.3390/molecules29204851/s1, Table S1: Volatile flavor components in different stages of Daqu production; Table S2: The α-diversity index of bacteria in medium-temperature Daqu at different processing stages; Table S3: The α-diversity index of Fungus in medium-temperature Daqu at different processing stages; Table S4: Core operational taxonomic units (OTUs) and their relative content in all Daqu samples of this study; Figure S1: Shannon dilution curve of bacteria (A) and fungi (B) in Daqu.
