# Supplementary material for: Dynamic Changes and Potential Correlations between Microbial Diversity and Volatile Flavor Compounds in Chinese Medium-Temperature Daqu during Manufacturing
Source: Molecules. 2024 Oct 13;29(20):4851. doi: 10.3390/molecules29204851 (PMC11509951; doi:10.3390/molecules29204851)
Supplement: Supplementary file 1 [file molecules-29-04851-s001.zip › molecules-3208443-supplementary.pdf]

Table S1. Volatile flavor components in different stages of Daqu production.

Name of VOCs	Category of VOCs	CAS	Relative content/%			
			DA	DB	DC	DD
1-Hexanol-M	Alcohols	111-27-3	2893.1±88.78	668.88±15.79	488.29±23.76	2019.53±20.5
1-Hexanol-D		111-27-3	486.97±58.43	192.06±14.31	206.32±13.33	263.76±34.36
1-Pentanol-M		71-41-0	4753.32±91.42	3105.32±258.28	342.62±35.55	566.62±8.35
1-Pentanol-D		71-41-0	1555.42±22.5	377.16±7.62	140.41±20.03	172.27±17.69
3-Methylbutanol-M		123-51-3	7852.25±373.13	7377.11±197.9	2859.76±896.4	5554.86±421.7
3-Methylbutanol-D		123-51-3	8362.8±392.54	12627.59±1968.78	1328.96±639.5	3159.72±504.08
Butanol-M		71-36-3	1110.76±43.73	1166.28±132.52	590.44±43.02	984.52±20.62
Butanol-D		71-36-3	204.98±7.97	149.72±11.32	86.4±12.85	106.03±3.83
1-Propanol		71-23-8	2136.54±25.11	1568.01±49.25	1034.1±51.84	463.81±3.87
Butan-2-ol		78-92-2	434.05±18.63	983.71±50.3	140.84±11.89	113.93±4.45
2-Methyl-1-propanol-M		78-83-1	3333.22±123.22	3785.06±178.81	2649.55±102.2	2379.65±56.94

2-Methylpropanol-D		78-83-1	3156.37±76.24	4409.14±1145.95	847.41±61.98	768.23±154.1
3-hydroxybutan-2-one		513-86-0	11683.23±758.84	2796.68±160.07	1191.27±26.86	998.57±14.88
3-Hydroxy-2-butanone		513-86-0	19292.6±2353.98	2142.74±165.48	900.6±161.85	750.48±20.54
1-hydroxypropan-2-one		116-09-6	2949.76±163.64	1557.84±181.78	465.99±88.32	440.25±55.66
Cyclohexanone		108-94-1	3861.59±504.07	11333.14±399.45	5815.46±107.36	4238.76±275.78
Cyclopentanone-M		120-92-3	276.26±30.02	3934.55±309.2	371.4±33.16	384.68±27.45
Cyclopentanone-D	Ketones	120-92-3	558.42±16.7	4004.81±621.67	267.99±52.2	291.08±25.29
2-heptanone		110-43-0	93.34±7.99	346.55±42.76	77.92±14.28	59.19±6.41
heptan-2-one		110-43-0	982.7±69.08	1749.12±46.69	387.62±30.58	493.24±30.48
3-penten-2-one, 4-methyl		141-79-7	202.78±3.86	159.86±16.98	43.29±1.31	158.01±18.48
Pentan-2-one		107-87-9	2949.68±207.31	2312.04±173.48	303.46±34.43	374.25±7.88
3-Methyl-2-butenal		107-86-8	616.59±24.41	1032.85±56.91	636.13±77.7	779.94±14.29
Hexanal-M	Aldehydes	66-25-1	2602.49±88.11	1096.87±167.23	734.75±65.8	1294.42±182.39
Hexanal-D		66-25-1	1951.35±184.96	535.41±40.5	309.69±34.81	528.73±113.36

acrolein		107-02-8	2069.88±105.17	1948.5±35.9	1749.3±20.38	1624±27.53
octanal		124-13-0	98.41±17.06	285.98±37.49	96.82±10.56	75.37±6.41
butanal-M		123-72-8	9510.31±128.55	7531.47±597.2	5004.8±334.55	4177.5±113.22
Hexanal		66-25-1	102.21±26.06	714.94±82.75	72.45±12.32	74.61±8.78
butanal-D		123-72-8	303.21±21.94	956.52±82.69	371.63±13.49	574.03±76.18
Butanoic acid propyl ester		105-66-8	589.61±58.32	191.9±46.16	108.09±20.31	101.65±5.48
Methyl butyrate		623-42-7	2695±194.41	866.92±118.67	472.1±56.99	633.54±43.28
ethyl 3-methylbutanoate-M		108-64-5	197.13±20.37	1163.13±110.01	50.74±6.42	59.37±9.82
ethyl 3-methylbutanoate-D	Esters	108-64-5	312.55±4.96	1375.61±64.23	126.31±28.28	160.2±2.68
Ethyl 2-methylbutanoate		7452-79-1	1111.99±73.85	606.59±14.58	188.02±26.4	90.89±5.38
ethyl acetate-M		141-78-6	383.83±71.8	541.86±68.54	270.4±52.21	229.29±22.41
ethyl acetate-D		141-78-6	10061.77±316.21	1992.51±198.53	278.34±12.27	188.42±14
2-Ethyl-6-methylpyrazine	Pyrazines	13925-03-6	3180.05±685.11	8919.93±1105.61	9463.32±2865.74	10530.52±1007.6
2,5-Dimethylpyrazine		123-32-0	164.45±38.27	2883.63±145.26	253.39±60.97	447.44±19.43

2,2,4,6,6-Pentamethylheptane-M	Hydrocarbons	13475-82-6	1578.46±66.29	1130±148.56	832.37±84.78	476.59±58.58
2,2,4,6,6-Pentamethylheptane-D		13475-82-6	1570.86±82.56	3253.68±134.27	2354.37±75.1	1788.14±246.53
beta-pinene-M		127-91-3	1128.64±43.84	1090.62±22.46	481.69±28.07	1406.62±88.4
beta-pinene-D		127-91-3	646.92±59.56	656.48±72.78	133.64±7.72	179.23±16.17
Limonene		138-86-3	453.18±26.47	2082.99±95.21	484.79±12.92	381.86±23.4
Acetic acid-M	Acids	64-19-7	12006.14±1673.38	6350.9±1157.47	3732.72±240.37	6085.1±331.24
Acetic acid-D		64-19-7	2275.92±789.44	2131.66±353.97	1716.55±476.59	2297.52±433.37
Butan-2-one-M	Others	78-93-3	445.42±13.4	315.71±26.99	517.64±107.13	400.79±20.29
Butan-2-one-D		78-93-3	2499.78±107.31	3285.78±112.36	1885.39±132.57	878.79±7.82
Ethenylbenzene		287399-41-1	741.05±39.61	848.71±28.93	867.73±2.25	298.4±16.24
2-Pentylfuran		3777-693	714.18±45.25	3449.96±622.86	119.89±8.98	260.34±26.53

Table S2. The α-diversity index of bacteria in medium-temperature Daqu at different processing stages.

	Shannon	Simpson	Chao1	ACE	Sobs	Coverage
DA	1.815	0.265	98.528	100.491	92.333	1.000
DB	1.514	0.387	71.367	72.747	67.000	1.000
DC	2.397	0.182	130.29	130.689	119.333	1.000
DD	1.498	0.434	89.643	92.611	86.333	1.000

Table S3. The α-diversity index of Fungus in medium-temperature Daqu at different processing stages.

	Shannon	Simpson	Chao1	ACE	Sobs	Coverage
DA	2.268	0.167	71.333	70.932	70.000	1.000
DB	0.761	0.519	21.556	30.902	15.667	1.000
DC	1.525	0.272	57.619	48.809	39.333	1.000
DD	0.593	0.666	17.500	18.017	15.667	1.000

Table S4. Core operational taxonomic units (OTUs) and their relative content in all Daqu samples of this study.

Kingdom	OTUs ID	Mean relative content	genus
Bacteria	OTU88	24.16%	*Weissella*
	OTU100	20.38%	*Thermoactinomyces*
	OTU90	17.24%	*Kroppenstedtia*
	OTU86	10.22%	*Staphylococcus*
	OTU94	4.65%	*Lactobacillus*
	OTU105	3.95%	*norank*
	OTU96	3.20%	*Lactobacillus*
	OTU95	2.47%	*Lactobacillus*
	OTU78	2.01%	*Lactobacillus*
	OTU223	1.64%	*Pantoea*
	OTU106	1.43%	*Lactobacillus*
	OTU98	1.13%	*Bacillus*
	OTU101	1.10%	*Leuconostoc*
	Others	6.42%	
Fungi	OTU121	36.09%	*Thermomyces*

	OTU117	27.31%	*Thermoascus*
	OTU126	12.13%	*Aspergillus*
	OTU43	7.94%	*Aspergillus*
	OTU133	6.56%	*Alternaria*
	OTU124	5.75%	*Saccharomycopsis*
	OTU120	2.11%	*Aspergillus*
	Others	2.11%	

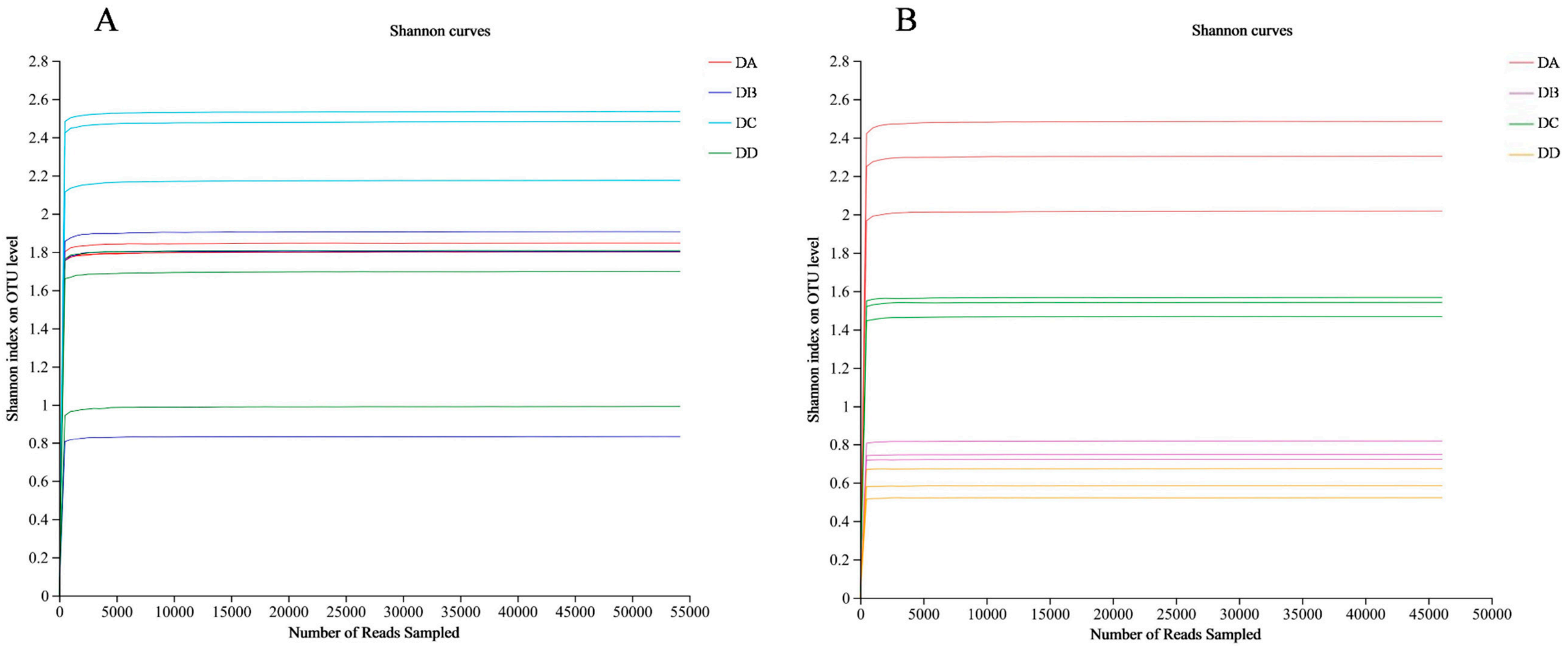

Fig. S1. Shannon dilution curve of bacteria (A) and fungi (B) in Daqu.
